# Melatonin for pre-medication in children: a systematic review

**DOI:** 10.1186/s12887-022-03149-w

**Published:** 2022-02-24

**Authors:** Katie Mellor, Diana Papaioannou, Anna Thomason, Robert Bolt, Chris Evans, Matthew Wilson, Chris Deery

**Affiliations:** 1 School of Health and Related Research, University of Sheffield, Sheffield, UK; 2 School of Clinical Dentistry, University of Sheffield, Sheffield, UK; 3 Department of Applied Health Research, University College London, London, UK

**Keywords:** Melatonin, Children, Pre-medication, Surgery, Anxiety

## Abstract

**Background:**

Melatonin’s effectiveness as an anxiolytic medication has been confirmed in adults; however, its efficacy in a paediatric population is unclear. A number of small studies have assessed its use in children as a pre-operative anxiolytic, with conflicting results.

**Methods:**

We undertook a systematic review of pre-operative melatonin use in children. Four databases (MEDLINE, Embase, the Cochrane Central Register of Controlled Trials and Web of Science), and ‘ClinicalTrials.gov’ were searched for ongoing and completed clinical trials of relevance. Citation tracking reference lists and relevant articles were also accessed. The review was unrestricted by comparator or outcomes. Eleven studies were judged eligible for inclusion. There were high levels of heterogeneity in melatonin administration (in terms of dose and timing). Variable outcomes were reported and included: anxiety; anaesthetic success; analgesia; sedation; post-operative recovery; and safety. Outcomes were not always assessed with the same measures.

**Results:**

Evidence to support melatonin’s anxiolytic properties in this setting is conflicting. Melatonin was associated with reduced sedative effects, post-operative excitement and improved emergence behaviour, compared to comparator drugs. One study reported the benefit of melatonin use on sleep disturbance at two weeks post-surgery. No adverse safety events were identified to be significantly associated with melatonin, affirming its excellent safety profile.

**Conclusion:**

Despite potential advantages, including improved emergence behaviour, based on current evidence we cannot confirm whether melatonin is non-inferior to current “usual care” pre-medications. Further consideration of melatonin as an anxiolytic pre-medication in paediatric surgery is needed.

**Supplementary Information:**

The online version contains supplementary material available at 10.1186/s12887-022-03149-w.

## Background

Melatonin is a natural sleep promoting neurohormone synthesised within the pineal gland. Aside from regulation of circadian rhythm, melatonin’s physiological functions include antioxidant, oncostatic, anti-inflammatory and anticonvulsant effects [1]. Melatonin can be produced synthetically. European Medicines Agency licensed tablets (2 mg Circadin®, UK and 3 mg Bio Melatonin, Hungary), and a 1 mg/ml oral solution exist (Colonis Pharma). Unlicensed liquid formulations are also available in the UK (Kidmel® & Kidnaps®, Special Products Limited, UK), as well as unlicensed 2-3 mg generic capsule formulations [2].

Melatonin is used in children and neonates to manage a number of conditions, including sleep and seizure disorders and neonatal sepsis. Melatonin has also been evaluated for its use as a pre-operative anxiolytic and has promising potential due to its reduced sedative effect compared to other anxiolytics. Melatonin’s anxiolytic properties are considered to be a consequence of its facilitatory role on γ-aminobutyric acid (GABA) transmission [3]. Although the effectiveness of melatonin as an anxiolytic pre-medication in adults has been confirmed through multiple clinical trials [4–10] and systematic reviews [11–13], its usefulness in a paediatric pre-operative setting is less certain. The existing literature has described an excellent safety profile; melatonin has no known major side effects and is well-tolerated [14].

Standard anxiolytic pre-medications in the paediatric setting include benzodiazepines, alpha2 agonists (clonidine or dexmedetomidine), and H1 antihistaminics [15]. Although effective, these drugs are associated with an increased sedative effect that may lengthen post-anaesthetic recovery. Melatonin offers a number of potential advantages, including ambulant rather than bed transfer to theatre, reduced post-operative sedation & sleep disturbance, faster recovery, improved post-operative analgesia, and avoidance of respiratory depression [16, 17]. In addition, some melatonin formulations may offer greater taste acceptance compared to the bitter flavour of conventional pre-medications, which could potentially improve compliance in a paediatric population. A number of small clinical trials have been conducted to assess melatonin pre-medication in the paediatric setting [18–28], although results are conflicting. Given the potential benefits melatonin has over alternative pre-medications, there is a need to determine whether there is an evidence base for its anxiolytic function in children.

In 2014, Andersen et al. [11] published a systematic review and meta-analysis of the efficacy and safety of peri-operative melatonin, finding a significant reduction in post-operative pain and pre-operative anxiety. The review drew from a general adult & child population, and since publication there has been a number of more recent randomised controlled trials conducted in a specifically paediatric population.

The primary aim of this systematic review is to determine the current evidence for the use of melatonin as a pre-operative anxiolytic in children.

## Methods

A protocol for this systematic review is available on PROSPERO (registration: CRD42018098940). The review has been conducted and reported according to PRISMA guidelines [29]. Randomised controlled trials (RCTs) were included with no restriction on comparator, outcomes, randomisation generation, blinding, publication date or language. The population was limited to children (aged 0–18 years). Studies evaluating any surgical intervention were considered eligible. Studies evaluating medical diagnostic procedures were excluded. No restriction was placed on melatonin formulation or dosage, trial comparator, or outcome. Four electronic databases were searched including MEDLINE, Embase, the Cochrane Central Register of Controlled Trials and Web of Science. ‘ClinicalTrials.gov’ was searched for ongoing and completed clinical trials. Backward citation tracking of reference lists, and forward citation tracking of relevant articles were also used. The search strategy is presented in Additional file 1.

Literature search exports were de-duplicated by KM using Elsevier Mendeley Desktop software [30], and confirmed by the built in ‘Check for Duplicates’ function. Two independent reviewers (KM and AT) considered the title and abstract of each study, excluding those not relevant. KM obtained the full texts of all studies that appeared eligible. KM and AT independently identified those studies for final inclusion. Where eligibility queries arose, guidance from a senior reviewer (DP) was sought.

A tailored data extraction form in Microsoft Excel was used to extract data. This was undertaken by KM and confirmed by AT. Data collected included: publication details, study design and characteristics; surgery details; anaesthesia details; intervention details (including melatonin dose, formulation, route of administration, and timing given); comparator details; any outcome measures and adverse events reported. The latest version (October 2018) of the revised Cochrane risk-of-bias tool for randomized trials (RoB 2.0) was used to assess bias of included studies [31]. This was undertaken independently by KM and AT. Any discrepancies were resolved by discussion with DP. Authors were contacted for clarification where information was omitted from the publication.

Summary measures are reported in a narrative synthesis. A meta-analysis was not appropriate in this study due to the small body of existing trials, and heterogeneity of the data. Findings with a P-value of <0.05 were considered to be significant and are described as such.

## Results

**Fig. 1:**
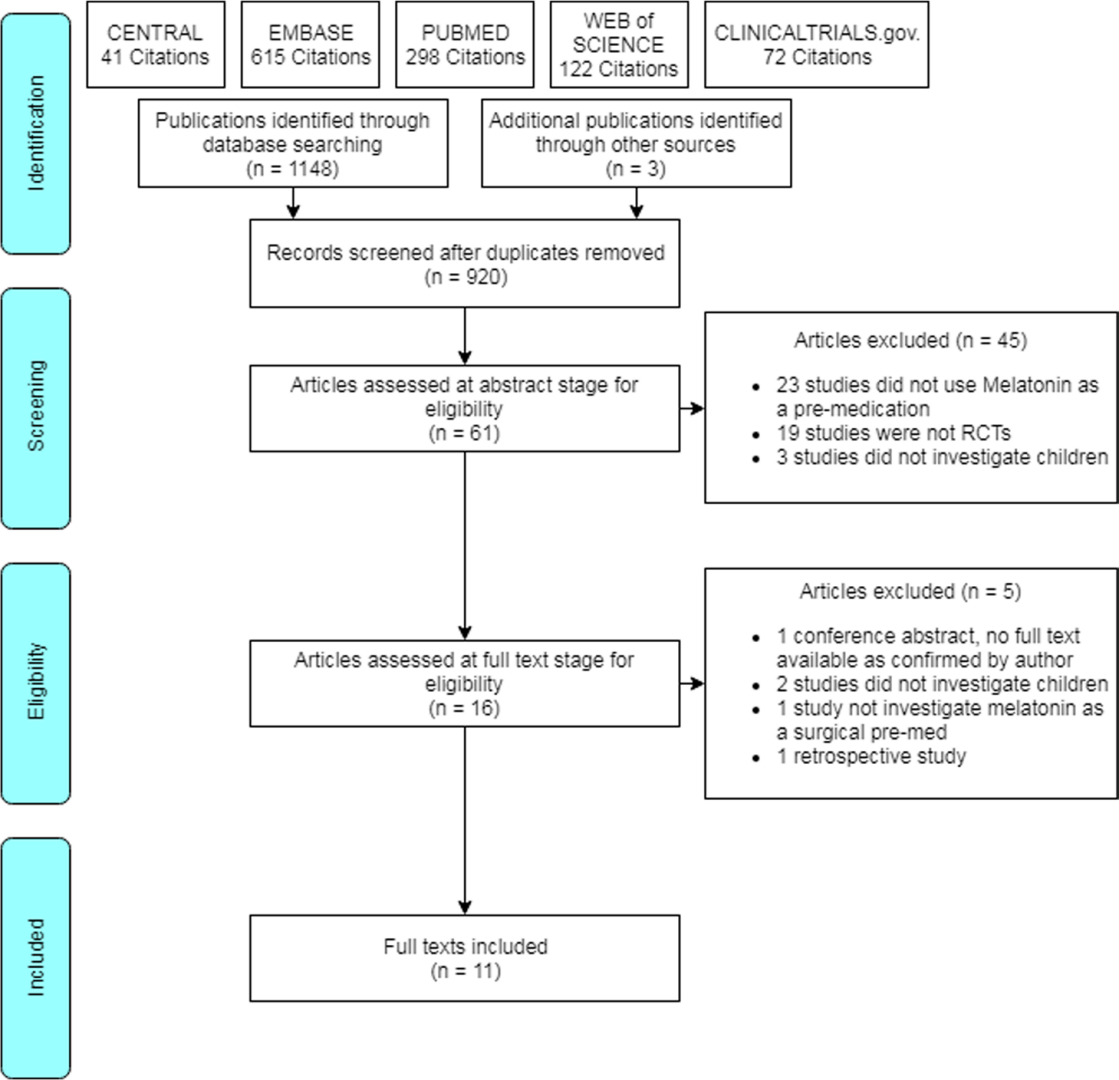
PRISMA flow chart

Electronic database searches retrieved 1148 results. Three additional results were obtained through citation tracking and screening reference lists. 920 results remained following deduplication. The abstracts of 61 articles were screened, of which 16 full text articles were assessed. Five of these were deemed ineligible due to either study design or population (i.e. did not investigate children), leaving eleven articles for inclusion in the review [18–28] (Fig. 1).

### Study characteristics

Included studies were published between 2005 and 2018, and were conducted in Italy, Iran, Turkey, USA, Egypt and India. Sample sizes ranged from 23 to 148 children and population age ranged from 1 to 15 years. Surgical populations included dental [18, 19, 28], adeno-tonsillectomy [24], oesophageal dilatation procedures [26], and other forms of minor elective surgery [21–23, 25, 27]. General anaesthesia was used in eight studies [19–24, 26, 27]. One study did not specify type of anaesthesia; the authors confirmed that both local and general anaesthesia were used [25]. Two studies used sedation without local anaesthesia [18, 28]. Three studies restricted inclusion criteria to an anxious population [18, 19, 28], defined as having negative/uncooperative behaviour according to the Frankl Behavioural Scale (FBS) [32]. Full study details are summarised in Table 1.

**Table 1:** Full Study Details

First author	Year	Country	n	Surgery / procedure	Age (years)	M:F	Anxious children	Anaesthesia	Melatonin dose	Melatonin formulation	Trade Name	Manufacturer	Preparation	Administration timing	Comparator(s)
Almenrader	2013	Italy	87	Not specified	1 - 5	77:10	No	GA	0.3 mg/kg	6mg/ml	Melamil®	Milte Italia SpA, Italy	Not specified	60 min prior to surgery	Clonidine 4μg/kg
Ansari	2018	Iran	23	Dental surgery	2 - 6	17:6	Yes	Sedation	0.5 mg/kg	Not specified	Not specified	Vitane Pharmaceuticals, USA	Dissolved in sweetened water	30 minutes prior to IV sedation	Midazolam 0.5 mg/kg
Faghihian	2018	Iran	132	Dental surgery	3 - 6	55:77	Yes	GA	0.5 mg/kg	15 ml	Melatonin tablets 3mg	Aristo pharmaceutical company, India	Dissolved in water	40 min prior to induction	Midazolam 0.5 mg/kg; Placebo (15 ml dextrose and saline)
Gitto	2016	Italy	92	Elective surgery	5 - 14	62:30	No	GA	0.5 mg/kg (max 20 mg)	Not specified	Melamil® oral drops	Milte Italia SpA, Italy	Dissolved in 5 ml water	40 min prior to induction	Midazolam 0.5 mg/kg (max 20 mg)
Impellizzeri	2017	Italy	80	Elective surgery	8 - 14	64:16	No	GA	0.5 mg/kg (max 20 mg)	Not specified	Melamil® oral drops	Milte Italia SpA, Italy	Dissolved in 5 ml water	40 min prior to induction	Midazolam 0.5 mg/kg (max 20 mg)
Isik	2008	Turkey	60	Dental surgery	3 - 8	31:29	Yes	Sedation	3mg; 0.5 mg/kg	Not specified	Melatonina® tablets (3mg)	Katowice, Poland	Dissolved in 3–10 ml water	60 min prior to N_2_O/O_2_ sedation	Midazolam 15mg/3ml; Placebo (saline)
Kain	2009	USA	148	Elective surgery	2 - 8	82:66	No	GA	0.05; 0.2; 0.4 mg/kg	Not specified	Not specified	Sigma Chemical, USA	Prepared in an investigational pharmacy	45 min prior to induction	Midazolam 0.5 mg/kg
Khalifa	2013	Egypt	120	Adenotonsillectomy	3 - 6	64:56	No	GA	0.1 mg/kg	Not specified	Not specified	Not specified	One tablet (5mg) dissolved in 10 ml glucose 5%. The calculated dose added with 15mg/kg oral paracetamol	60 min prior to induction	Ketamine 0.5 mg/kg; Melatonin 0.05 mg/kg with Ketamine 0.25 mg/kg; Placebo (saline)
Kurdi	2016	India	100	Elective surgery	5 - 15	51:49	No	LA and GA	0.5; 0.75 mg/kg	3mg/ml	Not specified	Life extension pharmaceuticals, Lauderdale, obtained online	Commercially available MT syrup (no preparation)	60 min prior to induction	Midazolam 0.5 mg/kg; Placebo (multivitamin syrup)
Ozcengiz	2011	Turkey	100	Esophageal dilatation procedures	3 - 9	50:50	No	GA	0.1 mg/kg	Not specified	Not specified	Not specified	Given with 2–2.5 mg/kg oral paracetamol	40–45 min prior to induction	Midazolam 0.5 mg/kg; Dexmedetomidine 2.5 μg/kg; Placebo (saline)
Samarkandi	2005	USA	105	Inguinal hernia, undescended testis, hydrocoele and hypospadias	2 - 5	73:32	No	GA	0.1; 0.25; 0.5 mg/kg	Not specified	Not specified	General Nutrition Corporation, USA	Mixed in 15mg/kg oral paracetamol	45 min prior to induction	Midazolam (0.1; 0.25; 0.5 mg/kg); Placebo (acetaminophen)

### Comparators

Ten studies compared melatonin to midazolam [18, 19, 21–28], either directly or amongst other arms including placebos [18, 19, 24, 25, 27] and dexmedetomidine [26]. One study compared melatonin with clonidine alone [20]. One study compared melatonin to ketamine, placebo, and a combination of melatonin and ketamine in half doses [24].

### Dose and formulation of melatonin

Three studies used Melamil® oral drops, [20–22] one Melatonina® tablets [18], and the remaining studies did not specify formulation or trade name. All but two studies provided manufacturer information [18–23, 25, 27, 28], which included companies in Italy, Poland, the USA and India. One study [25] noted that the Melatonin was obtained online (Life Extension pharmaceuticals). Two studies did not report manufacturer information [24, 26]. Melatonin preparation varied. Five studies mixed the active ingredient with water (ranging from 3 - 10 ml) [18, 19, 21, 22, 28], and three gave melatonin together with oral paracetamol [24, 25, 27]. One study used melatonin syrup, and therefore no preparation was required [25]. One study did not specify preparation [20]. All studies administered the medication orally. Dosing ranged from 0.05 mg/kg to 0.75 mg/kg, and two studies capped dosage at 20 mg [21, 22]. Three studies assessed melatonin effect using a dose range; Kain et al. trialled 0.05, 0.2 and 0.4 mg/kg [23], Kurdi et al. trialled 0.5 and 0.75 mg/kg [25] and Samarkandi et al. trialled 0.1, 0.25 and 0.5 mg/kg [27]. The timing of melatonin administration varied from 30–60 min prior to induction of anaesthesia. Intervention details are summarised in Table 1.

### Outcome measures

Five studies explicitly stated a primary outcome measure. These included success of steal induction (inhalational induction in a sleeping child) [20], effect on propofol requirements [21], pre-operative anxiety [22, 23], and anxiolysis, sedation, maintenance of cognition & psychomotor skills [25]. Additional reported outcomes included analgesia [19, 27], emergence behaviour [24, 26], duration of recovery [19, 20, 27], success of anaesthetic induction [19, 22, 23, 25], and adverse events [18, 20, 28]. Reported outcomes are summarised in Table 2.

**Table 2:** Outcomes reported

Outcome theme	Outcome reported	Almenrader	Ansari	Faghihian	Gitto	Impellizzeri	Isik	Kain	Khalifa	Kurdi	Ozcengiz	Samarkandi
**Anxiety**	Pre-operative anxiety (*n*=433)					**0**^a^		**-**^a^		**+**^a^		0
	Behaviour during separation from parents (*n*=100)									+		
	Post-operative anxiety (*n*=80)					0						
	Parental anxiety (*n*=228)					0		0				
**Anaesthesia**	Compliance to intravenous induction (*n*=460)			-		0		-		+		
	Successful steal induction (*n*=87)	**0**^a^										
	Quality of mask induction (*n*=87)	0										
	Required infusion of propofol (*n*=92)				**+**^a^							
**Sedation**	Sedation success (*n*=407)		-	-	0		-			**-**^a^		
	Time to onset of sleep (*n*=87)	0										
**Recovery**	Recovery duration (*n*=324)	0		-								+
	Recovery score (*n*=197)				0							0
	Emergence behaviour (*n*=368)							+	0		0	
	Hemodynamic variables (*n*=123)		0								0	
	Maintenance of cognition & psychomotor skills (*n*=100)									**+**^a^		
**Analgesia**	Post-operative analgesia (*n*=237)			0								+
**Long term follow-up**	Behaviour (2-week post op) (*n*=105)											+

^a^Explicitly stated to be the primary outcome; - Melatonin was less effective than comparator; + Melatonin was more effective than comparator; 0 Melatonin was equally as effective as comparator

### Risk of bias assessment

Bias was assessed in all RCTs as per the Cochrane risk of bias assessment tool v2.0 [31]. Methodological quality assessed included selection, performance detection, attrition and reporting bias (Table 3). All studies used a method of random sequence allocation; methods included computer-generated lists and random number tables. Methods of allocation concealment included the use of sealed envelopes and central computer generated allocation [19–27]. Ten studies were double blinded (both participants and outcome assessors blinded to allocation) [18, 19, 21–28]; however only four studies specified that the melatonin administrator was also blinded [21–23, 27]. One study was single blinded to outcome assessors [20]. Two studies did not state whether outcome data was available for all randomised participants [19, 21].

**Table 3:** Risk of bias assessment of six methodological domains as per the Cochrane risk-of-bias tool (v2.0)

	Random sequence generation (selection bias)	Allocation concealment (selection bias)	Blinding of participants (performance bias)	Blinding of IMP administrators (performance bias)	Blinding of outcome assessment (detection bias)	Incomplete outcome data (attrition bias)	Selective reporting (reporting bias)
Almenrader (2013)	+	+	–	–	+	+	+
Ansari (2018)	+	/	+	–	+	+	+
Faghihian (2018)	+	+	+	/	+	/	+
Gitto (2016)	+	+	+	+	+	/	+
Impellizzeri (2017)	+	+	+	+	+	+	+
Isik (2008)	+	/	+	/	+	+	+
Kain (2009)	+	+	+	+	+	+	+
Khalifa (2013)	+	+	+	/	+	+	+
Kurdi (2016)	+	+	+	–	+	+	+
Ozcengiz (2011)	+	+	+	/	+	+	+
Samarkandi (2005)	+	+	+	+	+	+	+

+ Low risk of bias; / unspecified; − High risk of bias

Eight studies were designed to test an a priori proposed difference in treatment effect between the study interventions i.e. they were designed as superiority trials. In general, sample sizes were based on large treatment effects. Two studies provided complete details on sample size calculation, including an explanation for the selected difference in treatment effect [20, 23]. Six studies, although providing detail on sample size parameters, did not justify why they had selected a particular treatment effect [18, 22, 24–27]. Three studies failed to provide any details on how the sample size had been calculated [19, 21, 28].

### Outcomes Explored

#### Anxiety

Pre-operative anxiety was assessed in four studies (total 433 children) [22, 23, 25, 27]. Tabular data was available in all studies, with three also presenting graphical data [23, 25, 27]. No studies reported significantly different baseline anxiety levels between trial arms.

All studies used the Modified Yale Pre-operative Anxiety Scale (mYPAS) to assess pre-operative anxiety [33]. The Spielberger State-Trait Anxiety Inventory for Children (STAI-C) [34] was used in one study to assess child anxiety the day before surgery [22]. Assessment time points varied between studies and were either specified by event, e.g. ‘day before surgery’ [22]; ‘before pre-medication’ [25, 27]; ‘in the pre-operative room’ [22]; ‘separation from parents’ [25, 27]; or numerically, e.g. ‘45 minutes prior to induction’; ‘10, 30, 45, 60 minutes following pre-medication’; and ‘10 minutes post-operative’ [22, 23, 25, 27].

There is conflicting evidence for the use of melatonin as a pre-operative anxiolytic in children. Kurdi et al. supported the use of melatonin (0.5 and 0.75 mg/kg) to decrease pre-operative anxiety, with the higher dose (0.75 mg/kg) reported to be most effective [25]. Impellizzeri et al. and Samarkandi et al. concluded melatonin (0.25 and 0.5 mg/kg) to be equally as effective as comparators in reducing anxiety [22, 27]. Kain et al. reported that children who received melatonin at a range of lower dosages (0.05, 0.2 and 0.4 mg/kg) were significantly more anxious compared to comparator, with no significant difference between doses [23]. In addition, all four studies assessing anxiety related outcomes, did not study a specifically anxious population, thereby potentially diluting any observable effects as an anxiolytic compared to either active or placebo control.

Two studies assessed parental anxiety prior to surgery [22, 23] using the STAI [34]. One study assessed anxiety of mothers only [22]. In both studies parental anxiety did not differ between the two arms. One study did identify a statistically significant correlation between mother’s and child’s anxiety in both trial arms [22]. Where parental anxiety was measured at different time points during the preoperative period, there was a significant increase in anxiety at later time points such as at separation from the child in both arms, with no significant association to melatonin dose or comparator [23].

#### Anaesthetic induction

Evidence for the effect of melatonin on induction compliance (total 460 children), was conflicting [19, 22, 23, 25]. One study found no significant difference between melatonin and midazolam against the Induction Compliance Checklist (ICC) [22], another concluding significantly lower ICC in children pre-medicated with melatonin compared to midazolam (50 vs 73%) [23]. In terms of IV access, Faghihian et al. reported that midazolam was superior (statistically significant) to melatonin on ease of IV access, and melatonin was not statistically different to placebo [19]. On the contrary Kurdi et al. reported greatest venepuncture compliance at the highest dose of melatonin, although results were not statistically significant [25].

There was no significant difference between melatonin compared to clonidine for the performance of steal induction (clonidine was effective in 13% more children), and the efficacy of melatonin was found to be dependent on the time of day administered. No age-dependent effect of melatonin was observed [20]. Gitto et al. investigated the effect of melatonin pre-medication, compared to midazolam, on propofol infusion requirements. The study concluded that melatonin significantly reduced the overall dosage of propofol infusion [21].

#### Sedation

Melatonin’s sedative effect prior to anaesthesia was assessed in five studies (total 407 children). All studies used different measures of sedation success including a unreferenced Sedation Scale [19], the Ramsay Sedation Scale (RSS) [18, 35], an Observers Sedation Scale (OSS) [25, 36], a Houpt Sedation Rating Scale [28, 37] and the University of Michigan Sedation Scale (UMSS) [21, 38]. Four studies reported that melatonin did not contribute towards sedation prior to anaesthesia, was similar to placebo, and was inferior to comparators [18, 19, 25, 28]. Three/four studies investigated a specifically anxious population (according to the Frankl Behavioural Scale (FBS)) [18, 19, 28]. Gitto et al. reported that patients who had received melatonin were equally as sedated as those who had received midazolam [21]. Based on these five studies there is some evidence for melatonin’s reduced sedative effect in this setting.

#### Analgesia

Two studies reported pain associated outcomes. Faghihian et al. found melatonin to reduce post-operative pain compared to placebo (quantified as analgesic requirements, of any modality, to discharge) [19]. The effect of melatonin compared to midazolam on need for analgesics is unclear, as the tabular data conflicts with the text. Samarkandi et al. reported melatonin to be superior to midazolam in reducing post-operative excitement (assessed using the modified pain/discomfort scale at 10 min post-operative). As the authors state, this scale does not differentiate between pain and excitement. In addition, all children received a caudal block and paracetamol which confounds evidence to support any analgesic benefit [27]. Based on these studies there is limited clinical evidence to support the analgesic effect of melatonin.

#### Recovery

Emergence behaviour was reported for 368 children. Kain et al. reported a statistically significant reduction in emergence delirium with melatonin compared to midazolam, as assessed by the Keegan scale [23, 39]. Two studies reported significant reductions in emergence agitation compared to placebo, with the reduction being similar to other comparators [24, 26].

Two studies evaluated post-operative recovery using the Aldrete Scale [40, 41]; both reported no significant difference between melatonin and comparator [21, 27]. Only one study investigated a longer-term follow up outcome post-discharge. Samarkandi et al. assessed sleep disturbance two weeks post-operatively using the Post Hospitalisation Behaviour Questionnaire (PHBQ) [42], reporting that melatonin pre-medication was associated with a significantly lower incidence of sleep disturbance compared to midazolam [27].

Recovery duration was assessed in three studies (total 324 children) and was conflicting. Almenrader et al. reported no significant difference between melatonin and clonidine in time to discharge [20]. Faghihian et al. reported that patients who had received melatonin had a significantly longer recovery (defined as time to discharge, decided by the anaesthetist based on the modified Aldrete criteria) than those who had received midazolam [19]. Samarkandi et al. reported that melatonin was associated with a faster recovery compared to midazolam (defined as scoring eight on the modified Aldrete scale), although this result was not statistically significant. Samarkandi also noted a trend in increased midazolam dose and protracted recovery duration. This trend was not present for melatonin [27]. There is overall conflicting evidence for the effect of melatonin pre-medication on recovery outcomes.

#### Safety profile

Adverse events (AEs) were reported in three studies [18, 20, 28], and three further studies explicitly stated that there were no associated AEs [25–27]. Where AEs were not mentioned in the publication the authors were contacted, with three further authors confirming that no AEs were recorded throughout the duration of the study [21, 22, 28]. AEs were rarely associated with melatonin use. Two studies recorded post-operative nausea and vomiting, cough and hiccough within both melatonin and comparator groups [18, 20]. One study reported a significantly lower incidence of nausea and vomiting, tremors and dizziness in children pre-medicated with melatonin compared to midazolam [28]. In further support of melatonin’s safety profile, two studies reported no significant difference between melatonin and comparators on pre-operative and intra-operative hemodynamic variables, including heart rate, electrocardiogram, blood pressure and oxygen saturation [26, 28]. Kurdi et al. assessed the effect of melatonin on cognitive and psychomotor function and found no impact, whereas the comparator, midazolam was significantly associated with cognition and psychomotor dysfunction [25]. The evidence supports melatonin’s safety profile in children.

## Discussion

### Summary of evidence

This review identified eleven studies conducted in the pre-operative paediatric setting comparing melatonin with alternative pre-medications. The wide variability in dosing of melatonin, comparators, outcomes and outcome measures used in each study, and inconsistent a priori sample size calculation has limited the ability to draw any definitive conclusion to support or refute melatonin’s use as a pre-operative anxiolytic medication. The conflicting results between studies might be in part due to inconsistencies in population, and the dose/formulation of melatonin used. Most studies (eight/eleven) did not study a specifically anxious population. This might reflect a range of practices between different healthcare settings. Within the UK, anaesthetists make the clinical decision on a case-by-case basis as to whether a child should receive a pre-medication for anxiety, whereas in other health care settings routine pre-medication is often standard.

Evidence to support reduction of anxiety and improvement/equivalence of anaesthetic success is conflicting. Four/five included studies indicate that melatonin has a reduced sedative effect compared to comparator [18, 19, 25, 28]. No study found melatonin to be inferior on recovery-associated outcomes [21, 23, 24, 26, 27], including the longer-term outcome, reduced sleep disturbance at 2 weeks post-operatively [27]. Kurdi et al. identified a dose-dependent effect of melatonin for alleviating pre-operative anxiety in children, with higher doses being more effective [25]. Kain et al. identified a dose-dependent effect of melatonin on improving emergence delirium [23]. These results are consistent with a systematic review and meta-analysis of emergence agitation in children who underwent general anaesthesia, concluding melatonin premedication to be effective in preventing emergence agitation, with increased dose significantly correlating with effect [43]. Improvement in recovery outcomes has potential impact of reduced resource use, faster anaesthetic turnaround times, and improved patient and carer centred outcomes e.g. reduced sedation and emergence dysphoria, and improved compliance.

The review confirms melatonin’s excellent safety profile as a pre-medication in children, with very few adverse events recorded and attributable to melatonin. Midazolam, a frequent comparator in the included studies, is a benzodiazepine and is an effective paediatric pre-operative medication [44]. Midazolam has been associated with some adverse effects including sedation and delayed post-operative recovery [45, 46]. Further concerns include the potential for respiratory suppression [47], and unpredictable effects which may result in agitation rather than anxiolysis, particularly in children with additional needs [48]. The NPSA 2008 rapid response document highlights the risk of overdose associated with bolus dosing of midazolam in adults [49], so it is reasonable to also identify alternative pre-medications for the paediatric setting. An alternative pre-medication, clonidine, has also been associated with adverse events including bradycardia, hypotension and prolonged recovery [50].

### Limitations

Inclusion of a small number of studies overall (total 1047 patients), with large degrees of heterogeneity, preclude a network meta-analysis, therefore studies cannot be combined. The small sample sizes of included studies (max n = 148), also suggests that the available evidence may not be sufficiently powered to detect a desired magnitude of treatment effect. Examination of the sample size justifications in the studies confirmed this, with nine studies failing to provide a complete justification for the sample size used. This ranged from providing no details on the sample size calculation [19, 21, 28] to failing to justify the treatment effect to be tested [18, 22, 24–27].

There is clinical difficulty, especially in children, to differentiate between sedation and anxiolysis. The natural sleep-inducing properties of melatonin can produce apparent sedation, enabling anaesthetic induction, without knowing whether sufficient anxiolysis has been obtained. This may have confounded findings in included studies and should be a consideration of future designs to include specifically anxious populations.

### Implications for practice/future research

For future trials, consideration should be given as to whether superiority trial designs are appropriate. The included studies appeared to be designed to test a superiority of treatment effect, whereas for many outcomes superiority of melatonin might not be necessary. A non-inferiority trial design, where an agreed difference in treatment effect is considered not clinically significant, might be preferable if sufficient improvements in other outcomes such as adverse effects or faster anaesthetic turnarounds and reduced resource use, can be demonstrated.

Inconsistencies regarding dose, administration timing, and formulation, outline a need for future trials and pharmacokinetic studies to produce clearer guidance regarding the optimal administration of melatonin as a paediatric pre-medication. Studies within this review suggest that melatonin is more effective at higher doses of 0.5 to 0.75 mg/kg [25], and less effective at lower doses [23]. Melatonin administration time ranged from 30–60 min prior to induction (no timing windows reported). This administration schedule is generally consistent with a recent systematic review of the pharmacokinetics of melatonin in adults, which reported the time to maximum melatonin concentration following oral immediate release formulation as 50 min [51].

Included studies trialled varied forms of melatonin. Omission of the trade name/manufacturer of melatonin from a number of studies raises concerns over the quality assurance of the active ingredient trialled. Future trials should use melatonin produced under Good Manufacturing Practice (GMP) [52].

## Conclusion

This systematic review details the current evidence for the use of pre-operative melatonin in children. There is a clear need for more rigorous, larger scale randomised controlled trials to assess the effectiveness of melatonin as a pre-operative medication. Future studies should use a quality assured melatonin product, consider the outcomes to be studied and the statistical design; including a properly justified sample size. The authors of this review are currently involved in a UK wide large, non-inferiority, randomised controlled trial of 624 anxious children aged 5–14, which will try to determine whether melatonin is efficacious in this setting [53].

## Supplementary Information

**Additional file 1.** Electronic search strategy

## Data Availability

The datasets used and/or analysed during the current study are available from the corresponding author on reasonable request. Additional file 1 includes all search strategies.

## Abbreviations

AE: Adverse Event
FBS: Frankl Behavioural Scale
GMP: Good Manufacturing Practice
ICC: Induction Compliance Checklist
PROSPERO: International prospective register of systematic reviews
IV: Intravenous
IB: Investigator Brochure
MHRA: Medicines and Healthcare products Regulatory Agency
mYPAS: Modified Yale Pre-operative Anxiety Scale
NHS: National Health Service
OSS: Observers Sedation Scale
PHBQ: Post-Hospital Behaviour Questionnaire
PRISMA: Preferred Reporting Items for Systematic Reviews and Meta-Analyses
RSS: Ramsay Sedation Scale
RCT: Randomised Controlled Trial
RoB: Risk of Bias
STAI: Spielberger State-Trait Anxiety Inventory
STAI-C: Spielberger State-Trait Anxiety Inventory for Children
SmPC: Summary of Product Characteristics
UMSS: University of Michigan sedation scale
