# Supplementary material for: Melatonin for pre-medication in children: a systematic review
Source: BMC Pediatr. 2022 Feb 24;22:107. doi: 10.1186/s12887-022-03149-w (PMC8876113; doi:10.1186/s12887-022-03149-w)
Supplement: Supplementary file 1 — Additional file 1. Electronic search strategy [file 12887_2022_3149_MOESM1_ESM.docx]

**Additional file 1** Electronic search strategy

**Embase (accessed via OVID – dating back to 1974) – 29-May-2019**

| # | Query | Items |
| --- | --- | --- |
| 1 | children.ti,ab,kw. | 1214797 |
| 2 | Pediatric*.ti,ab,kw. | 418261 |
| 3 | paediatric*.ti,ab,kw. | 106108 |
| 4 | 1 or 2 or 3 | 1470171 |
|  |  |  |
| 5 | Melatonin.ti,ab,kw. | 28900 |
|  |  |  |
| 6 | Perioperative.ti,ab,kw. | 122895 |
| 7 | Postoperative.ti,ab,kw. | 577885 |
| 8 | Preoperative.ti,ab,kw. | 315020 |
| 9 | Surgery.ti,ab,kw. | 1508343 |
| 10 | Operation.ti,ab,kw. | 414933 |
| 11 | premed*.ti,ab,kw. | 14314 |
| 12 | pre-med*.ti,ab,kw. | 1662 |
| 13 | anxiolytic*.ti,ab,kw. | 19780 |
| 14 | anxiety.ti,ab,kw. | 251300 |
| 15 | Anti-Anxiety.ti,ab,kw. | 1326 |
| 16 | analgesic*.ti,ab,kw. | 110805 |
| 17 | Pain.ti,ab,kw. | 854715 |
| 18 | Sleep.ti,ab,kw. | 228289 |
| 19 | Hypnotic*.ti,ab,kw. | 17521 |
| 20 | Sedative*.ti,ab,kw. | 25838 |
| 21 | 6 or 7 or 8 or 9 or 10 or 11 or 12 or 13 or 14 or 15 or 16 or 17 or 18 or 19 or 20 | 3261323 |
|  |  |  |
| 22 | 4 and 5 and 21 | 664 |
| 23 | limit 22 to human | 615 |

**Medline – 29-May-2019**

| # | Query | Items |
| --- | --- | --- |
| 1 | children[MeSH Terms] | [1830571](https://www.ncbi.nlm.nih.gov/pubmed/?cmd=HistorySearch&querykey=1) |
| 2 | Pediatrics[MeSH Terms] | [55303](https://www.ncbi.nlm.nih.gov/pubmed/?cmd=HistorySearch&querykey=2) |
| 3 | paediatric[Title/Abstract] | [53732](https://www.ncbi.nlm.nih.gov/pubmed/?cmd=HistorySearch&querykey=3) |
| 4 | Pediatrics[Title/Abstract] | [33605](https://www.ncbi.nlm.nih.gov/pubmed/?cmd=HistorySearch&querykey=4) |
| 5 | children[Title/Abstract] | [972550](https://www.ncbi.nlm.nih.gov/pubmed/?cmd=HistorySearch&querykey=5) |
| 6 | OR (1-5)  ((((children[MeSH Terms]) OR Pediatrics[MeSH Terms]) OR paediatric[Title/Abstract]) OR Pediatrics[Title/Abstract]) OR children[Title/Abstract] | [2140020](https://www.ncbi.nlm.nih.gov/pubmed/?cmd=HistorySearch&querykey=6) |
|  |  |  |
| 7 | Melatonin[MeSH Terms] | [18706](https://www.ncbi.nlm.nih.gov/pubmed/?cmd=HistorySearch&querykey=7) |
| 8 | Melatonin[Title/Abstract] | [22947](https://www.ncbi.nlm.nih.gov/pubmed/?cmd=HistorySearch&querykey=8) |
| 9 | OR (7-8)  (Melatonin[Title/Abstract]) OR Melatonin[MeSH Terms] | [24613](https://www.ncbi.nlm.nih.gov/pubmed/?cmd=HistorySearch&querykey=9) |
|  |  |  |
| 10 | Perioperative Period[MeSH Terms] | [82075](https://www.ncbi.nlm.nih.gov/pubmed/?cmd=HistorySearch&querykey=10) |
| 11 | Perioperative[Title/Abstract] | [84717](https://www.ncbi.nlm.nih.gov/pubmed/?cmd=HistorySearch&querykey=11) |
| 12 | Postoperative Period[MeSH Terms] | [51875](https://www.ncbi.nlm.nih.gov/pubmed/?cmd=HistorySearch&querykey=12) |
| 13 | Postoperative[Title/Abstract] | [439856](https://www.ncbi.nlm.nih.gov/pubmed/?cmd=HistorySearch&querykey=13) |
| 14 | Preoperative Period[MeSH Terms] | [6123](https://www.ncbi.nlm.nih.gov/pubmed/?cmd=HistorySearch&querykey=14) |
| 15 | Preoperative[Title/Abstract] | [231947](https://www.ncbi.nlm.nih.gov/pubmed/?cmd=HistorySearch&querykey=15) |
| 16 | surgery[MeSH Subheading] | [1965439](https://www.ncbi.nlm.nih.gov/pubmed/?cmd=HistorySearch&querykey=16) |
| 17 | Surgery[Title/Abstract] | [1148622](https://www.ncbi.nlm.nih.gov/pubmed/?cmd=HistorySearch&querykey=17) |
| 18 | surgical procedures, operative[MeSH Terms] | [2983488](https://www.ncbi.nlm.nih.gov/pubmed/?cmd=HistorySearch&querykey=18) |
| 19 | Operation[Title/Abstract] | [319512](https://www.ncbi.nlm.nih.gov/pubmed/?cmd=HistorySearch&querykey=19) |
| 20 | premedication[MeSH Terms] | [25405](https://www.ncbi.nlm.nih.gov/pubmed/?cmd=HistorySearch&querykey=20) |
| 21 | pre-med*[Title/Abstract] | [868](https://www.ncbi.nlm.nih.gov/pubmed/?cmd=HistorySearch&querykey=21) |
| 22 | premed*[Title/Abstract] | [10882](https://www.ncbi.nlm.nih.gov/pubmed/?cmd=HistorySearch&querykey=22) |
| 23 | anxiolytics[MeSH Terms] | [18246](https://www.ncbi.nlm.nih.gov/pubmed/?cmd=HistorySearch&querykey=23) |
| 24 | anxiety[Title/Abstract] | [174192](https://www.ncbi.nlm.nih.gov/pubmed/?cmd=HistorySearch&querykey=24) |
| 25 | Anti-Anxiety Agents[MeSH Terms] | [18246](https://www.ncbi.nlm.nih.gov/pubmed/?cmd=HistorySearch&querykey=25) |
| 26 | analgesics[MeSH Terms] | [181982](https://www.ncbi.nlm.nih.gov/pubmed/?cmd=HistorySearch&querykey=26) |
| 27 | pain[MeSH Terms] | [375239](https://www.ncbi.nlm.nih.gov/pubmed/?cmd=HistorySearch&querykey=27) |
| 28 | Pain Relief[Title/Abstract] | [29456](https://www.ncbi.nlm.nih.gov/pubmed/?cmd=HistorySearch&querykey=28) |
| 29 | (Hypnotics and Sedatives[MeSH Terms]) | [28503](https://www.ncbi.nlm.nih.gov/pubmed/?cmd=HistorySearch&querykey=29) |
| 30 | Sleep[MeSH Terms] | [75014](https://www.ncbi.nlm.nih.gov/pubmed/?cmd=HistorySearch&querykey=30) |
| 31 | OR (10-30)  ((((((((((((((((((((Perioperative Period[MeSH Terms]) OR Perioperative[Title/Abstract]) OR Postoperative Period[MeSH Terms]) OR Postoperative[Title/Abstract]) OR Preoperative Period[MeSH Terms]) OR Preoperative[Title/Abstract]) OR surgery[MeSH Subheading]) OR Surgery[Title/Abstract]) OR surgical procedures, operative[MeSH Terms]) OR Operation[Title/Abstract]) OR premedication[MeSH Terms]) OR pre-med*[Title/Abstract]) OR premed*[Title/Abstract]) OR anxiolytics[MeSH Terms]) OR anxiety[Title/Abstract]) OR Anti-Anxiety Agents[MeSH Terms]) OR analgesics[MeSH Terms]) OR pain[MeSH Terms]) OR Pain Relief[Title/Abstract]) OR ((Hypnotics and Sedatives[MeSH Terms]))) OR Sleep[MeSH Terms] | [4792202](https://www.ncbi.nlm.nih.gov/pubmed/?cmd=HistorySearch&querykey=31) |
|  |  |  |
| 32 | 6 AND 9 AND 31  (((((((children[MeSH Terms]) OR Pediatrics[MeSH Terms]) OR paediatric[Title/Abstract]) OR Pediatrics[Title/Abstract]) OR children[Title/Abstract])) AND ((Melatonin[Title/Abstract]) OR Melatonin[MeSH Terms])) AND (((((((((((((((((((((Perioperative Period[MeSH Terms]) OR Perioperative[Title/Abstract]) OR Postoperative Period[MeSH Terms]) OR Postoperative[Title/Abstract]) OR Preoperative Period[MeSH Terms]) OR Preoperative[Title/Abstract]) OR surgery[MeSH Subheading]) OR Surgery[Title/Abstract]) OR surgical procedures, operative[MeSH Terms]) OR Operation[Title/Abstract]) OR premedication[MeSH Terms]) OR pre-med*[Title/Abstract]) OR premed*[Title/Abstract]) OR anxiolytics[MeSH Terms]) OR anxiety[Title/Abstract]) OR Anti-Anxiety Agents[MeSH Terms]) OR analgesics[MeSH Terms]) OR pain[MeSH Terms]) OR Pain Relief[Title/Abstract]) OR ((Hypnotics and Sedatives[MeSH Terms]))) OR Sleep[MeSH Terms]) | [298](https://www.ncbi.nlm.nih.gov/pubmed/?cmd=HistorySearch&querykey=32) |

**Cochrane Library – 29-May-2019**

| # | Query | Items |
| --- | --- | --- |
| 1 | MeSH descriptor: [Child] explode all trees | 1158 |
| 2 | MeSH descriptor: [Pediatrics] explode all trees | 628 |
| 3 | "paediatric":ti,ab,kw (Word variations have been searched) | 28028 |
| 4 | #1 or #2 or #3 in Trials | 29139 |
|  |  |  |
| 5 | MeSH descriptor: [Melatonin] explode all trees | 1071 |
| 6 | "melatonin":ti,ab,kw (Word variations have been searched) | 2416 |
| 7 | #5 or #6 | 2416 |
|  |  |  |
| 8 | "perioperative":ti,ab,kw (Word variations have been searched) | 16437 |
| 9 | "postoperative":ti,ab,kw (Word variations have been searched) | 104261 |
| 10 | "preoperative":ti,ab,kw (Word variations have been searched) | 32136 |
| 11 | "surgery":ti,ab,kw (Word variations have been searched) | 189912 |
| 12 | MeSH descriptor: [General Surgery] explode all trees | 333 |
| 13 | "operation":ti,ab,kw (Word variations have been searched) | 32816 |
| 14 | "premedication":ti,ab,kw (Word variations have been searched) | 7495 |
| 15 | MeSH descriptor: [Anti-Anxiety Agents] explode all trees | 2150 |
| 16 | "anxiety":ti,ab,kw (Word variations have been searched) | 43166 |
| 17 | MeSH descriptor: [Analgesics] explode all trees | 19536 |
| 18 | MeSH descriptor: [Pain] explode all trees | 44197 |
| 19 | "pain relief":ti,ab,kw (Word variations have been searched) | 13808 |
| 20 | MeSH descriptor: [Hypnotics and Sedatives] explode all trees | 3479 |
| 21 | MeSH descriptor: [Sleep] explode all trees | 5287 |
| 22 | 8 or 9 or 10 or 11 or 12 or 13 or 14 or 15 or 16 or 17 or 18 or 19 or 20 or 21 | 1245936 |
| 23 |  |  |
| 24 | #4 and #7 and #22 in Trials | 41 |

**Clinicaltrials.gov – 29-May-2019**

Intervention: Melatonin

Population: Children

= 72 studies

**Web of Science – 29-May-2019**

| # | Query | Items |
| --- | --- | --- |
| 1 | TOPIC: (Pediatrics) OR TOPIC: (Paediatrics) OR TOPIC: (Child*) | 1,768,653 |
|  |  |  |
| 2 | TOPIC: (Melatonin) OR TITLE: (Melatonin) | 33,127 |
|  |  |  |
| 3 | TOPIC: (perioperative) OR TOPIC: (postoperative) OR TOPIC: (preoperative) OR TOPIC: (surgery) OR TOPIC: (operation) OR TOPIC: (premed*) OR TOPIC: (pre-med*) OR TOPIC: (anxiety) OR TOPIC: (Anxiolytic*) OR TOPIC: (Analgesic*) OR TOPIC: (Pain) OR TOPIC: (Hypnotic*) OR TOPIC: (Sedative*) OR TOPIC: (Sleep) | 3,227,364 |
|  |  |  |
| 4 | #3 AND #2 AND #1 | 804 |
|  |  |  |
| 5 | ALL=(clinical trial) | [845,477](https://apps.webofknowledge.com/summary.do?product=WOS&doc=1&qid=13&SID=F6GGMn64cU6SY3ZBaBT&search_mode=AdvancedSearch&update_back2search_link_param=yes) |
|  |  |  |
| 6 | #5 AND #4 | [122](https://apps.webofknowledge.com/summary.do?product=WOS&doc=1&qid=14&SID=F6GGMn64cU6SY3ZBaBT&search_mode=CombineSearches&update_back2search_link_param=yes) |
